# Combined Use of Job Stress Models and the Incidence of Glycemic Alterations (Prediabetes and Diabetes): Results from ELSA-Brasil Study

**DOI:** 10.3390/ijerph17051539

**Published:** 2020-02-27

**Authors:** Raíla de Souza Santos, Rosane Härter Griep, Maria de Jesus Mendes da Fonseca, Dóra Chor, Itamar de Souza Santos, Enirtes Caetano Prates Melo

**Affiliations:** 1 Department of Epidemiology and Quantitative Methods in Health, National School of Public Health, Oswaldo Cruz Foundation, 21041-210 Rio de Janeiro, Brazil; mariajmf@ensp.fiocruz.br (M.d.J.M.d.F.); dorachor@fiocruz.br (D.C.); enirtes@globo.com (E.C.P.M.); 2 Laboratory of Health and Environment Education, Oswaldo Cruz Institute, Oswaldo Cruz Foundation, 21040-360 Rio de Janeiro, Brazil; 3 Center of Clinical and Epidemiological Research, University Hospital, University of São Paulo (USP), 05508-000 São Paulo, Brazil; itamarss@usp.br

**Keywords:** occupational stress, demand–control model, effort–reward imbalance, prediabetes state, diabetes mellitus type 2, health status disparities

## Abstract

Evidence of psychosocial stress at work as a risk factor for diabetes and prediabetes is restricted. Objectives: Analyze the independent and combined association of the models, demand–control and social support (DC-SS) and the effort–reward imbalance and overcommitment (ERI-OC), and the incidence of glycemic alterations (prediabetes and diabetes). Methods: A prospective study was carried out with data from 7503 active workers from the Brazilian Longitudinal Study of Adult Health (ELSA-Brasil) study in the period 2008–2014. Work stress was measured by two stress models. Glycemic levels were evaluated by glycated hemoglobin (HbA1c) in two moments and classified in four groups: normal, maintenance of prediabetes, incident prediabetes, and incident diabetes. Multinomial logistic regression was analyzed with 5% significance levels stratified by sex, and multiplicative interactions were investigated. Results: Work stress and glycemic alterations were more frequent in women. Psychosocial stress at work was shown to be associated to the risk of prediabetes and diabetes only among women. For women, the combination of models enlarged the magnitude of the association: prediabetes (DC-ERI = OR 1.51, 95% CI 1.15–1.99) and diabetes (DC-ERI = OR 2.10, 95% CI 1.20–3.65). Highly-educated women exposed to ERI-OC were four times more likely to have diabetes. Conclusion: Both models may contribute to explaining the psychosocial stress load according to each pattern of glycemic alteration among women.

## 1. Introduction

Diabetes mellitus type 2 (DM2), characterized by resistance to insulin, is responsible for approximately 90% of diabetes cases. DM2 is a chronic disease of great magnitude globally [1,2], and presents considerable growth of estimates in countries with low and middle incomes [3]. It is estimated that 60% of DM2 risk is due to modifiable behavior factors such as obesity, physical inactivity, quality of diet, and smoking [4]. Therefore, identifying other risk factors related to those cited, or that directly affect blood glucose, is relevant for preventing the disease.

Stress is one of the most relevant psychosocial factors in the development of diabetes; it affects blood glucose directly through neuroendocrine mechanisms (catecholamine, glucocorticoids, and inflammation biomarkers) that result in alterations in the production of hepatic glucose, insulin sensibility, and secretion [5,6], and maintains indirect action related to negative coping through disease risk behaviors [6,7]. From the perspective that stress has multiple etiologies, emphasis has been given to the work environment since economically active adults spend a considerable amount of their time in labor activities that are often considered stressful.

Although causal relations between occupational stress and cardiovascular diseases are well established, and diabetes represents one of the main risk factors for this specific outcome [8], there is restricted evidence, with contradictory results, on the relation between occupational stress and diabetes, and evidence is scarce for prediabetes [9]. Sectional studies carried out in Sweden, Belgium, Germany, and China [9,10,11,12], a case-control study in Sweden [13], and European longitudinal studies with different cohorts [14], as well as Swedish [15], English [7,16,17], German [18,19], Canadian [20], and American [21] studies showed that stress at work was positively associated to diabetes. 

However, some prospective studies carried out in the USA [22,23] and Japan [24], one sectional study in France [25], and two meta-analyses [26,27] did not confirm this association. Conversely, two longitudinal studies carried out in Israel [28] and a French study with three cohorts, a Swedish, and British study [29], did not find an association for high tension at work, only social support at work. 

Two important theoretical models are used to evaluate the deleterious effects of stress in work environments on health: demand–control and social support (DC-SS) model and the effort–reward imbalance and overcommitment (ERI-OC) model [30,31]. In association with diabetes studies there is, in the international literature, a prevalence of the use of model DC [7,13,14,16,20,25,26,32]. Few have used the ERI model [12,17,21]. Only Kumari’s study [17] used both models to evaluate the relation between stress and diabetes, but with isolated dimensions of stress models.

Models DC and ERI capture different aspects of psychosocial stress at work. While DC addresses those related to the psychosocial work environment, ERI considers, in addition, the organizational issues and aspects of work in the context of a more current global economy, intrinsic characteristics of worker perception, and the role of society in the process of recognizing this subject [33]. As measuring the psychosocial work environment is complex, recent recommendations suggest the combined use of models to evaluate health outcomes related to occupational stress [34,35,36]. Despite the lack of evidence to support the combined use of stress models (DC and ERI) for diabetes, studies have shown the increased predictive power of the combination, compared to results for each separate model, for self-reported health outcomes [35], insomnia [36], absenteeism [37], and cardiovascular problems [38].

Due to still-inconclusive evidence of the association of psychosocial stress at work and diabetes, the scarcity of studies on intermediary glycemic conditions (prediabetes) and the absence of studies that explore a combination of stress models to evaluate diabetes, the objectives of this study are to: (i) investigate the independent association of each component of the models (DC-SS and ERI-OC) and the maintenance of prediabetes as well as the incidence of prediabetes and diabetes; (ii) evaluate if the combination of models (DC-SS, ERI-OC, and DC-ERI) increases the predictive value of psychosocial stress at work, compared to the results of each model separately.

## 2. Materials and Methods

### 2.1. Design and Study Participants

This is a longitudinal analysis of the baseline data (2008–2010) and the second phase (2012–2014) of the Brazilian Longitudinal Study of Adult Health (ELSA-Brasil). The population of this multicenter study were civil servants from five universities and one research institute in six Brazilian capitals (15,105 participants in the baseline and 14,014 participants in the second wave) [39]. A more detailed description of the methodological aspects of ELSA-Brasil, such as data collection, clinical and laboratorial measures, and quality control procedures, can be found in Aquino and collaborators [39].

To be considered eligible for the analysis of this article, participants had to be active workers who followed in the study’s second wave (10,034) and completed information on occupational stress (91 participants who did not answer stress at work questionnaires were excluded). Additionally, 1207 participants diagnosed with DM at baseline were excluded according to the following criteria: self-referred diabetes or use of medicine for diabetes or glycated hemoglobin ≥6.5% or glucose tolerance test ≥200 mg/dL. From the total of an eligible 8736, 1154 presented a reduction in glycemic levels and 79 individuals presented missing data in the covariates used, totalizing an analytical sample of 7503 participants (3998 women and 3505 men).

### 2.2. Exposure Variable: Psychosocial Stress at Work

**Demand–control and social support:** obtained from the Brazilian version [40] of the Swedish demand–control and social support questionnaire developed by Theorell [41] that is based on the Job Content Questionnaire [30]. This instrument is composed of psychological demands (five items) that involve workload and psychological demands as well as job control (decision latitude six items). Four items refer to skill discretion and two to decision authority. In the case of both the demands and the job control scales, the response options are presented on a Likert-type scale (1–4), varying between “often” and “never/hardly ever”.

Each score was calculated separately: for demand the first tertile (low) was used as the reference category; for job control, the third tertile (high). We divided the scores obtained on the psychological demands scale by the one obtained on the job control scale and then divided the resulting ratio into tertiles. The highest tertile represented the ‘‘high job strain’’ group, according to the demand–control model, whereas the first (lowest) tertile ‘‘low job strain’’ was used as the reference category in the analyses. The use of tertiles makes this scale comparable to the ERI scale, which is most often divided into tertiles.

The dimension social support at work (SS) was also evaluated as a model and contains six questions with four response options on a Likert scale (1–4) from “totally agree” to “totally disagree” and was also categorized in tertiles, with the third tertile (high) as the reference category.

**Effort–reward imbalance and overcommitment (ERI-OC):** was measured by the Brazilian version [42] of the questionnaire designed by Siegrist [31], which assumes that work stress is generated by the imbalance between two components: high effort and low rewards [31]. The short version used in ELSA-Brasil is operationalized by 22 items distributed in three scales, in which participants indicated their degree of agreement with the statements (“strongly disagree” to “strongly agree”) on a four-point scale. 

The model contains two scales measuring extrinsic components: effort (six items), generated from external demands required at work, and reward (ten items, excluding one item on stability at work, as it is a stable cohort of civil servants) that considers financial gain (five items), perspective of promotion at work (four items), safety (one item), and recognition for work. In addition to these dimensions, the model has an intrinsic component, overcommitment (six items), considered as the factor that expresses a pattern of excessive effort in combination with a strong desire to be approved and esteemed. For each participant, a ratio was constructed using the formula: e/(r*c), of the effort–reward imbalance by the equation of the sum of the effort scores (e) divided by the sum of the reward scores (r) multiplied by the correction factor (c = 0.6), considering the number of numerator items compared to the denominator (6/10) [43].

All dimensions were categorized in tertiles. In the component “effort”, the first tertile (low) was used as a reference category; in the component “reward”, the third tertile (high) was used as a reference category; in the score obtained by the ratio between the two components (effort and reward) the first tertile was used as a reference category (low); in the component “overcommitment”, the first tertile (low) was considered as a reference category. The division in tertiles was adopted in order to enable comparison between the isolated dimensions of both models, according to other studies.

**Combinations between models:** three combinations of models/scales were tested—DC and SS, ERI and OC, and DC and ERI. For each combination, the respondents were divided into four exposure categories: those not exposed to any stressor according to the relevant model/scale (reference group); those exposed only to one stressor model; those exposed only to the other stressor model; and those simultaneously exposed to stressors according to both models. The division in tertiles was also adopted for the combination of models to establish comparability. Higher tertiles represent higher levels of psychosocial stress at work; the first tertile (absence of psychosocial stress) was used as a reference category.

### 2.3. Dependent Variable: Glycemic Levels, Prediabetes, and Diabetes Type 2

The maintenance and incidence of prediabetes, as the incidence of diabetes type 2, were defined by glycated hemoglobin (HbA1c) and measured in two moments of the study: time 1 (T1: 2008–2010) and time 2 (T2: 2012–2014). Analyses were carried out in a central laboratory to maintain a uniform quality control standard of exams. HbA1c was classified in accordance to the American Diabetes Association (2017) in two categories: “prediabetes” (HbA1c 5.7–6.4%) and “diabetes” (HbA1c ≥6.5%). 

Considering the two measurements of HbA1c during the follow-up period (T1 and T2), glycemic alterations were sub-categorized as: “normal” for participants with normal glycemic levels in both time moments (T1 and T2 HbA1c <5.7%); “maintenance prediabetes” for participants who maintained altered glycemic levels in both phases (T1 and T2 HbA1c 5.7–6.4%); “incident prediabetes” for participants who developed prediabetes in the second study wave (T1 HbA1c <5.7% and T2 HbA1c 5.7–6.4%); and “incident diabetes” for participants who developed diabetes in the second study wave (T1 HbA1c <6.5% and T2 HbA1c ≥6.5%).

### 2.4. Co-Variables

Sociodemographic characteristics were sex, age (continuous and dichotomized in 35–49 and 50 years up), and schooling (up to complete high school and complete higher education). Work characteristics were composed by weekly workload (up to 40 h/week and more than 40 h/week), work shift (daytime or nighttime), and by the occupational category based in science and technology concepts in three strata (manual, medium and higher). Healthy lifestyle habits, such as smoking (never smoked, ex-smoker, and smoker) and leisure physical activity, were evaluated by the long version of the International Physical Activity Questionnaire (IPAQ) that contemplates the type of activity and its intensity (strong, moderate, and weak). The body mass index (BMI) represents adiposity and was evaluated as a continuum of risk, calculated by the ratio of weight (kg) and squared height (kg/m^2^), and categorized for the descriptive analysis as “thinness or normal” (BMI lower than 24.9), overweight (BMI between 25 to 29.9), and obese (BMI equal or higher than 30).

### 2.5. Data Analysis

Analyses were stratified by sex, based on prior knowledge in the literature, according to which occupational stress and glycemic alterations differ between men and women. For descriptive analysis of the covariates we used the Chi-squared test (Pearson’s Chi-squared test) and *T*-test of the characteristics of the study population, the categories of psychosocial stress at work, and the distribution of incidence of glycemic alterations.

We used causal diagrams, in this case represented by a directed acyclic graph (DAG), in order to identify the set of co-variables included in the analyses [44]. Based on this strategy, schooling, age, and weekly workload and work shift were considered potential confounders. Once the confounders were proportionate to a “support path” between the exposure and outcome, the occupational category can be substituted by schooling (Figure S1). Physical activity, BMI, and smoking were considered mediators in this association, therefore they were not adjusted since the regression models used cannot capture the effect of mediation, and adjusting would remove the effect the study was trying to capture. Confounding variables representing the total effect of the relation between stress and diabetes were selected for adjustment (age, education, weekly workload, and work shift) (Figure S1).

Multinomial logistic regression models were estimated in three steps to evaluate the associations between exposure variables (psychosocial stress at work by both models DC-SS and ERI-OC) and the multinomial outcome (glycemic levels categorized as normal, maintenance prediabetes, incident prediabetes, and incident diabetes). An odds ratio (OR) and confidence interval of 95% were estimated for the crude model (crude OR) and for the models that were adjusted for age, education, shift, and weekly workload (adjusted ^A^ OR). 

The first step evaluated the association between each isolated dimension of the models: psychological demands, job control, demand-control, social support at work for the DC model; effort, reward, effort–reward imbalance, and overcommitment at work for the ERI model. The second stage analyzed the association between each complete model (DC and SS; ERI and OC) and the outcome and the association between the combination of partial models (DC and ERI) with the outcome. For each combination, groups were categorized into four exposure categories: not exposed to any stress model (reference category); exposed in a stressful model; exposed in another stressful model; and exposed to both models. For example, in the combination of DC-ERI, the four categories were: (i) those exposed to neither DC nor ERI (reference group); (ii) those exposed only to DC; (iii) those exposed only to ERI; and (iv) those exposed to both high DC and high ERI.

Finally, in the third stage we evaluated the modifying effect of schooling in the association of interest on the multiplicative scale for the combined models (DC and SS; ERI and OC; DC and ERI). All analyses were carried out using the free access R i386 free software, version 3.3.1 (The R Foundation for Statistical Computing, Institute for Statistics and Mathematics Wirtschaftsuniversit¨at Wien Welthandelsplatz, Vienna, Austria) and in the construction of DAGS we used DAGitty (Leeds Institute for Data Analytics, Leeds, UK and the Deutsche Forschungsgemeinschaft, Bonn, Germany) [44].

### 2.6. Ethical Aspects

The ELSA study was approved by the ethic committees of each institution involved and by the National Ethics Counsel in Research (CONEP). The present study was approved by the Research Ethics Committee of FIOCRUZ National School of Public Health (CAAE 656716.0.0000.5240).

## 3. Results

### 3.1. The Population under Study

In the population studied (Table 1), there was a predominance of women (53.3%); mean age was 51.3 ± 6.6 years for women and 52.0 ± 7.1 for men; women presented a higher schooling level (64.4%).

Regarding work-related factors, in the manual occupational category there was a predominance of men (25.8%). There were no differences according to sex regarding the distribution of work per shift; men reported a higher weekly workload. Relating to lifestyle, men had a higher frequency of smoking habits and overweight (46.7%) and women of obesity (26.0%) (Table 1).

According to our findings, higher exposure to occupational stress was observed among women. Likewise, for the simultaneous exposure to a combination of models, 24.9% of women and 16.7% of men were classified with a high DC ratio and low social support; 21.0% of women and 18.1% of men were classified with high ERI imbalance and high overcommitment; and finally, 25.7% of women and 19.0% of men were classified simultaneously for high DC ratio and high ERI imbalance (exposed in both partial stress models) (Table 2).

Glycemic alterations outcomes were observed more frequently among women. Similarly, women presented a higher frequency of job stress at work according to models analyzed in their isolated dimensions (Table 2). 

### 3.2. Association Between Psychosocial Stress by Isolated Models and Glycemic Alterations

Among men (Table 3), we did not observe a significant association for the dimensions of the demand–control and social support model after adjusting for confounding variables. Regarding the effort–reward model, only overcommitment was inversely proportional to the maintenance of prediabetes, with a dose-response gradient for those ranked in the highest exposure tertile (OR 0.64 95% CI 0.43-0.93) (Table 3).

For women (Table 4), regarding the demand–control and social support model, high psychological demands showed an association to the risk of diabetes (2nd tercile OR 2.27, 95% CI 1.26–4.09; 3rd tercile OR 2.41, 95% CI 1.30–4.50), with a sharp gradient standing out. Similarly, low control heightens the risk of prediabetes (2nd tercile OR 1.44, 95% CI 1.09–1.89; 3rd tercile OR 1.46, 95% CI 1.08–1.99). The combination of the two dimensions (demand–control ratio) showed that women classified in the higher tercile of exposure to occupational stress presented a 34% higher risk of prediabetes (95% CI 1.03–1.73) and 77% higher risk of diabetes (95% CI 0.98–3.19) compared to those classified in the lower tercile of exposure. The presence of low social support at work showed a higher risk of prediabetes (OR 1.29, 95% CI 0.95–1.75) and diabetes (OR 1.93, 95% CI 0.98–3.37) (Table 4). 

In analyses of the dimension of the effort–reward model, women exposed to low levels of reward at work presented a heightened risk for prediabetes (OR 1.26, 95% CI 0.97–1.64) and diabetes (OR 1.76, 95% CI 1.03–2.99). In women exposed to the higher tercile of overcommitment, a protection effect was observed, related to maintenance of prediabetes (OR 0.63, 95% CI 0.43–0.93); likewise this was found for men (Table 4).

### 3.3. Psychosocial Stress Analyzed by Means of Combinations of DC-SS, ERI-OC, and DC-ERI

The combination of the models with the inclusion of their dimensions (DC-SS and ERI-OC) and the combination of the partial models (DC-ERI) provided an increase in associative strength only for women (Table 5 and Table 6).

Among men (Table 5), an association with the maintenance of prediabetes was observed, but the significance was not maintained after adjustment for potential confounders.

Among women (Table 6), those classified with high DC and low SS (Model 1) presented a higher risk of prediabetes (OR 1.49, 95% CI 1.12–1.98) and independent association of DC exposure for prediabetes and diabetes (Table 6). For the combined exposure of high ERI and high OC (Model 2) in women, a higher risk of diabetes was observed (OR 1.82, 95% CI 1.07–2.07) and independent association, by the ERI model, for maintenance and higher risk of prediabetes (Table 6). 

Similarly, in women, the combined exposure of two models DC and ERI (Model 3) favored an increase in the strength of association for the incidence of prediabetes (OR 1.51, 95% CI 1.15–1.99) and diabetes (OR 2.10, 95% CI 1.20–3.65) (Table 6). A multiplicative interaction between schooling and ERI-OC was observed only for women (*p*-value 0.0371) (Table 6).

After adjustment for all co-variables, women with high schooling exposed to higher levels of psychosocial stress by ERI-OC (Model 2) presented a four times higher risk of diabetes (95% CI 1.96–9.60) (Table 7). Among women with low schooling, only increased risk of maintenance of prediabetes was observed over the period (Table 7).

## 4. Discussion

Our results show that psychosocial stress at work, measured by both stress models (DC and ERI) isolated and combined, was associated with a higher risk of prediabetes and diabetes among women. The combination of these stress models was more predictive than the isolated models. Schooling was shown as an important modifier in the associations observed in women. As far as we know, this is the first prospective study to combine two evaluation models of stress at work and evaluate their association to different patterns of glycemic alterations (prediabetes and diabetes).

The feminine over-risk related to job stress and its association to diabetes was also confirmed in other studies [7,10,11,12,13,15,16,20]. However, two important prospective studies found no association to women: Nurse’s Health Study II in the USA [23] and Whitehall II in England [17]. The first included only one specific professional group (nurses), with lower variability in the stress patterns at work, and the second a smaller number of women than men. Some studies showed a reduction in the risk of diabetes among men [7,15].

In the Brazilian context, domestic work is attributed almost exclusively to women, intensifying gender inequalities [39]. A study carried out with ELSA-Brasil data showed that women present a higher prevalence of conflict between work and family, and a lack of leisure time and personal care, when compared to men [45]. Women, while coping alone with a second workload, which is often more painful and exhausting, can be submitted to an over-risk, in which risk factors inherent to professional work add to those arising from domestic work, thus enhancing them [45]. This overload has harmful effects on physical health, mental health, and poorer self-rated health among women and its implications mark a higher risk of cardiovascular disease in studies that associate psychosocial stress in both occupational and family spheres [45,46,47].

Still regarding gender differences, in line with the literature, the results are in agreement with the DC model in which associations are predominate for women [7,10,11,13,15,16,20]; however, they diverge for the ERI model, which presents higher association of glycemic alterations for men [17,19]. Only one sectional study, carried out in China [12] with ERI, showed an association of heightened levels of glycated hemoglobin among women.

With reference to isolated models, there were differences between the dimensions of the DC model according to the level of glycemic alterations. The low job control may be the first dysfunction inducer; for that reason, it is associated to prediabetes in women. In the literature, low control is the dimension of highest impact for the occurrence of diabetes in women [10,11,13,15,20]. The relation with incident diabetes in our results was predominantly explained by the high psychosocial demand and by the ratio of the dimensions (demand–control) associated to the growth of both glycemic alterations (prediabetes and diabetes), which confirmed that the combination of dimensions has greater explanatory power, as presented in other studies [11,13,14,15,16,18]. For the ERI model domains, only low levels of work reward were associated to increased incident prediabetes and incident diabetes in women. Despite the restricted evidence for diabetes according to the ERI model, two transversal studies confirm our findings for low reward in women [9,12]. 

Regarding additional components of the models—social support and overcommitment—despite many studies excluding them from stress at work analyses [10,15,19,21], our results showed a relevant contribution of these components, both isolated and associated to the reference models (DC-SS and ERI-OC). 

The restriction of social support aggravates the stress at work situation, enlarging the negative effects on women’s health; on the opposite path, high social support can attenuate or counterbalance the effects of stress at work [48]. This impact may be identified for diabetes in two longitudinal studies in which low social support was the single component of the model associated to the risk of diabetes [28,29]; in addition, low social support was also associated to a higher resistance to insulin [49]. Similarly, in Norberg [13] and Heraclides [16], the impact of low social support for diabetes was only identified among women, corroborating our findings. 

For overcommitment, the inclusion of this component to the reference model (ERI-OC) generated a great impact for the risk of diabetes only in women. People characterized by overcommitment present attitudes, behaviors, and emotions that reflect exaggerated efforts, beyond levels considered appropriate. Studies confirm that overcommitment causes more damage in women [50], as seen for cardiovascular disease [38,51]; but no association to diabetes was found. To date, this is the first study to identify the association of overcommitment and diabetes in women.

Concerning the combination of the partial models DC and ERI, a higher magnitude of association for prediabetes and diabetes in women was found compared to the association found for these models when isolated. Given the conceptual differences, both models can contribute differently, explaining the stress load in accordance to each pattern of glycemic alteration. Work in the DC model is concentrated in task execution characteristics and is more related to situational aspects of the psychosocial environment; on the other hand, the ERI model concentrates on exchanging “costs” and “earnings” and includes extrinsic (related to the environment) and intrinsic (of the workers personal nature) situations. However, there is overlapping between both regarding the components’ “demand” and “effort”, which are identified in the results of the combination of models (DC-ERI) in the cases of incident diabetes.

At last, a modifying effect of schooling was observed, though in an opposite direction to the one expected: women with high schooling presented risk of glycemic alterations. As observed in our findings, in the study of Griep and collaborators [45] the schooling of women in ELSA-Brasil interacted with the conflict of work and family and self-reported health; women with high schooling presented a higher chance of worse self-reported health then those with lower schooling. This modifying effect was not found in men. A higher risk of diabetes in women with high schooling exposed to ERI-OC was found; no previous studies on the effect of schooling in the association of diabetes evaluated by the ERI model were found.

Some hypotheses that involve psychosocial environment characteristics of work can be raised on the higher risk of diabetes for the group of women with high schooling exposed to ERI-OC. First, the ERI model includes a larger number of stressing experiences related to work, such as satisfaction with salary, promotion possibilities, occupational and social self-esteem, and recognition of work, combined with overcommitment, which reflects a strong desire to be approved and esteemed [31]. 

All these characteristics represent common challenges that women from high social backgrounds that occupy more complex ranks and work positions face, and are subject to pressures and discriminations relative to gender in the construction of their careers. The situation seems to be aggravated by the growing demand for qualifications—a common characteristic in women with high schooling—in the work environment of universities and research institutes. These workers sometimes perform three different forms of work: professional, domestic, and educational [45]. Such elements could explain that schooling modifies the association between stress at work and the incidence of prediabetes and diabetes in women, but not in men. 

This prospective study made use of more than one criterion to identify individuals free of the disease—those without diagnosis of diabetes on the baseline (self-report or use of medication for diabetes or glycated hemoglobin or glycose tolerance oral test)—reducing the chance of reverse causality. It was possible to distinguish diabetes type 1 and type 2 by joining two pieces of information present on the baseline: age at diagnosis and the use of insulin as the first option for treatment. Few studies have evaluated gender differences for this research object. The use of two patterns of outcome, with border line changes in blood glucose, showed the impact of stress at work on the maintenance of glycemic alterations and in the incidence of prediabetes, an important condition for developing diabetes and cardiovascular diseases. Finally, the combined use of two theoretical models of stress at work, evaluated by questionnaires validated in Brazil which included social support and overcommitment components, offered an extensive basis of comparison.

Although the use of HbA1c may be underestimated for the occurrence of the outcome of interest (prediabetes and diabetes), it is a reliable measure of chronic blood glucose that presents technical advantages in analysis and predicts the progression of diabetes and cardiovascular events in a more consistent way [52]. In addition, a sensibility test, carried out for two prediabetes and diabetes outcome measurement criteria (isolated HbA1c or HbA1c and tolerance to glucose test), confirms that, for the diagnosis of diabetes and prediabetes, HbA1c emphasizes stress at work (data not presented). To date, HbA1c has been presented as an indicator of stress at work [53,54], both by the DC model [24,55] and ERI model [9,12,19].

Limitations should be considered, such as the use of DC by the ratio between psychological demands and control, since different values for the two dimensions can produce the same division result, that represents different situations of stress at work [56]. This option was adopted to allow comparisons with ERI, predominantly calculated by the ratio between the two components [57]; in addition, the DC model was analyzed in isolation. For the ERI model, the most common operationalization is the ratio between effort and reward, with results presented in tertiles [38,57]. The same model was adopted for this study. 

Lifestyle variables were considered mediators in the investigated association. It is important to notice that these variables represent different coping strategies to occupational stress and are considered risk behaviors for the development of diabetes. Individuals with higher levels of stress are more resistant to changes in lifestyle, such as healthy eating and physical activity, increasing sedentary lifestyle and obesity [4]. In this study, a similar proportion of men and women reported being smokers; obesity was more frequent among women, a determinant risk factor for our findings among women. It is possible that other potential mediators, not considered in the present analyses, also exist, such as other aspects of a low-quality diet and other factors of the work environment.

Another point to be considered is the fact that this study concentrated on stress in the work environment, which is the most studied form of occupational stress. However, there are other conceptualizations of stress-related works, such as work–family conflict, that could impact matters of gender not contemplated in this study [45], as well as other sources of stress that, despite happening outside the work environment, aggravate occupational stress. 

## 5. Conclusions

It was possible to conclude that both models, demand–control and effort–reward imbalance, may contribute to explaining the psychosocial stress load according to each pattern of glycemic alteration in women. 

The combination of DC and ERI showed a higher magnitude in the association for risk of prediabetes and diabetes when compared to isolated models and components. Social support at work and overcommitment were of great impact in evaluating the risk of prediabetes and diabetes in women, both isolated and associated to the reference model. 

Independent of the model used, a higher risk of prediabetes and diabetes was observed in women. Among men, significant associations were not observed between psychosocial stress at work and incidence of glycemic changes. 

It is believed that modifications in work relations should be rethought to promote higher control, autonomy, satisfaction, and recognition for women’s work to reduce occupational stress. Reducing overcommitment at work, which aggravates stress in the high schooling feminine group, is complex since it is an intrinsic component of the worker, but it is believed that higher gender equality in the work environment in women of high schooling who occupy more complex work positions would result in less of a need for overcommitment when seeking professional recognition. Therefore, gender equality should be a priority of occupational health policies in order to reduce gender discrepancies in domestic and professional work and to deliver tangible benefits, as seen in findings related to social support at work.

## Figures and Tables

**Table 1:** Sociodemographic, work, and lifestyle characteristics of the study sample, Brazilian Longitudinal Study of Adult Health (ELSA-Brasil) 2012–2014.

Character	Women	Men
	*n* (%)	*n* (%)
Total (7503)	3998 (53.3)	3505 (46.7)
Age		
Mean (standard deviation)	51.3 (6.6)	52.0 (7.1)
Schooling		
Up to complete high school	1423 (35.6) **	1482 (42.3) **
Complete undergraduate education	2575 (64.4)	2023 (57.7)
Occupational category		
Higher	1562 (39.2) **	1422 (40.5) **
Medium	2074 (51.8)	1179 (33.6)
Manual	362 (9.0)	904 (25.8)
Work shift		
Daytime	3622 (90.6)	3153 (90.0)
Nighttime	376 (9.4)	352 (10.0)
Weekly workload		
Up to 40 hours a week	1862 (46.6) **	1319 (37.6) **
More than 40 hours a week	2136 (53.4)	2186 (62.4)
Smoking habit		
Never smoked	2680 (67.0) **	2003 (57.2) **
Ex-smoker	920 (23.0)	1106 (31.5)
Smoker	398 (10.0)	396 (11.3)
Body mass index		
Thinness/normal	1499 (37.5) **	1101 (31.4) **
Overweight	1461 (36.5)	1636 (46.7)
Obese	1038 (26.0)	768 (21.9)

** *p* < 0.01 in the Chi-squared test for gender difference.

**Table 2:** Description of exposure and outcome variables of study sample, ELSA-Brasil 2012–2014.

Character	Women	Men
	*n* (%)	*n* (%)
Total (7503)	3998 (53.3)	3505 (46.7)
Exposure to Occupational Stress		
Demand–control model		
1st tertile (low score)	1192 (29.8) **	1350 (38.5) **
2nd tertile	1243 (31.1)	1146 (32.7)
3rd tertile	1563 (39.1)	1009 (28.8)
Social support		
1st tertile (high score)	761 (19.0) **	855 (24.4) **
2nd tertile	1791 (44.8)	1583 (45.2)
3rd tertile	1446 (36.2)	1067 (30.4)
Effort–reward model		
1st tertile (low score)	1222 (30.6) **	1172 (33.4) **
2nd tertile	1291 (32.3)	1192 (34.0)
3rd tertile	1485 (37.1)	1141 (32.6)
Overcommitment		
1st tertile (low score)	1064 (26.6) *	987 (28.2) *
2nd tertile	1660 (41.5)	1480 (42.2)
3rd tertile	1274 (31.9)	1038 (29.6)
Simultaneous Exposure to Occupational Stress		
DC and low SS present	997 (24.9) **	584 (16.7) **
ERI and OC present	838 (21.0) **	634 (18.1) **
DC and ERI present	1029 (25.7) **	667 (19.0) **
Glycemic Alterations Outcomes		
Normal	3248 (81.2) *	2944 (84.0) *
Prediabetes maintenance	256 (6.4)	185 (5.3)
Incident prediabetes	405 (10.1)	298 (8.5)
Incident diabetes	89 (2.2)	78 (2.2)

DC: demand–control model. SS: social support. ERI: effort–reward imbalance model. OC: overcommitment. * *p* < 0.05 and ** *p* < 0.01 in the Chi-squared test for gender difference.

**Table 3:** Multinomial logistic regression, with an odds ratio (OR) and confidence interval (CI) of 95%, of the association of psychosocial stress at work, measured by models DC-SS and ERI-OC, and adjusted glycemic alterations by selected variables, for active men in the second wave of ELSA-Brasil 2008–2014.

Men	Prediabetes Maintenance		Incident Prediabetes		Incident Diabetes	
	Crude OR (95% CI)	Adj OR ^A^ (95% CI)	Crude OR (95% CI)	Adj OR ^A^ (95% CI)	Crude OR (95% CI)	Adj OR ^A^ (95% CI)
Psychological Demands	**Demand–Control Model and Social Support at Work**					
1st tertile (low score)	1.0	1.0	1.0	1.0	1.0	1.0
2nd tertile	1.19 (0.85–1.66)	1.24 (0.89–1.74)	1.01 (0.77–1.33)	1.06 (0.80–1.40)	1.29 (0.78–2.15)	1.32 (0.79–2.21)
3rd tertile	0.97 (0.65–1.47)	1.09 (0.72–1.65)	1.08 (0.79–1.48)	1.19 (0.86–1.64)	1.14 (0.62–2.09)	1.22 (0.66–2.27)
Job Control						
1st tertile (high score)	1.0	1.0	1.0	1.0	1.0	1.0
2nd tertile	1.28 (0.90–1.83)	1.19 (0.82–1.73)	1.08 (0.81–1.44)	0.99 (0.73–1.33)	0.93 (0.54–1.59)	0.82 (0.47–1.44)
3rd tertile	1.37 (0.93–2.00)	1.11 (0.72–1.71)	1.38 (1.03–1.86)	1.14 (0.82–1.60)	1.21 (0.69–2.10)	0.95 (0.51–1.77)
Demand–Control						
1st tertile (low score)	1.0	1.0	1.0	1.0	1.0	1.0
2nd tertile	1.02 (0.71–1.46)	1.03 (0.71–1.48)	1.00 (0.75–1.33)	1.00 (0.75–1.34)	0.83 (0.48–1.45)	0.82 (0.47–1.42)
3rd tertile	1.22 (0.85–1.75)	1.16 (0.80–1.68)	1.11 (0.83–1.49)	1.05 (0.78–1.41)	1.10 (0.65–1.88)	1.02 (0.59–1.76)
Social Support						
1st tertile (high score)	1.0	1.0	1.0	1.0	1.0	1.0
2nd tertile	0.86 (0.60–1.22)	0.99 (0.69–1.42)	0.86 (0.64–1.15)	0.96 (0.71–1.3)	1.00 (0.56–1.78)	1.10 (0.61–1.98)
3rd tertile	0.67 (0.45–1.01)	0.82 (0.54–1.25)	0.81 (0.59–1.12)	0.95 (0.68–1.32)	1.11 (0.61–2.05)	1.29 (0.69–2.40)
Effort	**Effort–Reward Imbalance Model and Overcommitment**					
1st tertile (low score)	1.0	1.0	1.0	1.0	1.0	1.0
2nd tertile	0.81 (0.58–1.14)	0.87 (0.62–1.23)	0.84 (0.64–1.11)	0.86 (0.65–1.14)	0.93 0.55–1.57)	0.95 (0.56–1.62)
3rd tertile	0.72 (0.48–1.07)	0.82 (0.54–1.22)	0.95 (0.70–1.28)	1.04 (0.76–1.42)	1.05 (0.60–1.86)	1.14 (0.64–2.04)
Reward						
1st tertile (high score)	1.0	1.0	1.0	1.0	1.0	1.0
2nd tertile	1.05 (0.75–1.49)	1.08 (0.76–1.53)	1.14 (0.86–1.51)	1.14 (0.86–1.52)	0.76 (0.44–1.30)	0.76 (0.44–1.31)
3rd tertile	0.79 (0.53–1.17)	0.88 (0.59–1.32)	0.94 (0.68–1.28)	0.99 (0.72–1.36)	0.91 (0.52–1.58)	0.96 (0.55–1.68)
Effort–Reward Imbalance						
1st tertile (low score)	1.0	1.0	1.0	1.0	1.0	1.0
2nd tertile	0.84 (0.59–1.19)	0.91 (0.64–1.29)	0.85 (0.64–1.13)	0.89 (0.67–1.2)	0.85 (0.49–1.48)	0.89 (0.51–1.56)
3rd tertile	0.69 (0.48–1.00)	0.80 (0.55–1.17)	0.87 (0.65–1.16)	0.94 (0.70–1.27)	0.99 (0.58–1.71)	1.08 (0.62–1.88)
Overcommitment						
1st tertile (low score)	1.0	1.0	1.0	1.0	1.0	1.0
2nd tertile	0.71 (0.50–1.00)	0.72 (0.51–1.02)	0.97 (0.72–1.29)	1.01 (0.75–1.35)	0.83 (0.49–1.41)	0.86 (0.50–1.48)
3rd tertile	0.61 (0.41–0.90)	0.64 (0.43–0.96)	0.89 (0.65–1.22)	0.96 (0.69–1.34)	0.77 (0.43–1.38)	0.81 (0.44–1.49)

95% CI: 95% confidence interval. OR: odds ratio. Adjusted ^A^ crude model + adjusted by total effect variables (age—schooling level—weekly workload – work shift).

**Table 4:** Multinomial logistic regression, with an odds ratio (OR) and confidence interval (CI) of 95%, of the association of psychosocial stress at work, measured by models DC-SS and ERI-OC, and adjusted glycemic alterations by selected variables, for active women in the second wave of ELSA-Brasil 2008–2014.

Women	Prediabetes Maintenance		Incident Prediabetes		Incident Diabetes	
	Crude OR (95% CI)	Adj OR ^A^ (95% CI)	Crude OR (95% CI)	Adj OR ^A^ (95% CI)	Crude OR(95% CI)	Adj OR ^A^ (95% CI)
Psychological Demands	**Demand–Control Model and Social Support at Work**					
1st tertile (low score)	1.0	1.0	1.0	1.0	1.0	1.0
2nd tertile	0.84 (0.62–1.13)	0.89 (0.65–1.21)	1.09 (0.85–1.40)	1.09 (0.84–1.40)	2.24 (1.25–4.01)	2.27 (1.26–4.09)
3rd tertile	0.91 (0.66–1.25)	1.02 (0.73–1.43)	1.17 (0.90–1.53)	1.17 (0.89–1.54)	2.36 (1.29–4.33)	2.41 (1.30–4.50)
Job Control						
1st tertile (high score)	1.0	1.0	1.0	1.0	1.0	1.0
2nd tertile	1.35 (0.97–1.87)	1.21 (0.85–1.72)	1.38 (1.06–1.80)	1.44 (1.09–1.89)	0.90 (0.55–1.46)	0.87 (0.52–1.45)
3rd tertile	1.62 (1.16–2.27)	1.16 (0.79–1.71)	1.52 (1.16–2.01)	1.46 (1.08–1.99)	0.76 (0.44–1.32)	0.64 (0.34–1.18)
Demand–Control						
1st tertile (low score)	1.0	1.0	1.0	1.0	1.0	1.0
2nd tertile	0.86 (0.61–1.21)	0.84 (0.59–1.19)	1.00 (0.76–1.32)	0.96 (0.73–1.28)	1.87 (1.03–3.37)	1.79 (0.99–3.26)
3rd tertile	1.19 (0.88–1.62)	1.08 (0.79–1.49)	1.42 (1.10–1.82)	1.34 (1.03–1.73)	1.86 (1.04–3.30)	1.77 (0.98–3.19)
Social Support						
1st tertile (high score)						
2nd tertile	0.89 (0.64–1.25)	1.16 (0.82–1.64)	1.16 (0.86–1.55)	1.27 (0.94–1.70)	1.69 (0.86–3.29)	1.88 (0.96–3.70)
3rd tertile	0.80 (0.56–1.13)	1.10 (0.76–1.59)	1.16 (0.86–1.57)	1.29 (0.95–1.75)	1.69 (0.85–3.35)	1.93 (0.96–3.87)
Effort	**Effort–Reward Imbalance Model and Overcommitment**					
1st tertile (low score)	1.0	1.0	1.0	1.0	1.0	1.0
2nd tertile	0.94 (0.70–1.27)	1.13 (0.82–1.54)	1.05 (0.82–1.35)	1.12 (0.87–1.45)	0.95 (0.57–1.60)	1.02 (0.60–1.72)
3rd tertile	0.84 (0.61–1.15)	1.03 (0.73–1.45)	1.05 (0.81–1.36)	1.08 (0.82–1.42)	1.14 (0.68–1.90)	1.17 (0.68–2.01)
Reward						
1st tertile (high score)	1.0	1.0	1.0	1.0	1.0	1.0
2nd tertile	0.86 (0.63–1.17)	0.91 (0.66–1.26)	1.01 (0.78–1.31)	1.03 (0.80–1.34)	1.01 (0.58–1.76)	1.03 (0.59–1.81)
3rd tertile	1.01 (0.74–1.39)	1.22 (0.88–1.69)	1.17 (0.90–1.52)	1.26 (0.97–1.64)	1.60(0.94–2.70)	1.76 (1.03–2.99)
Effort–Reward Imbalance						
1st tertile (low score)	1.0	1.0	1.0	1.0	1.0	1.0
2nd tertile	0.86 (0.63–1.18)	1.02 (0.73–1.42)	0.94 (0.72–1.23)	1.01 (0.77–1.32)	0.72 (0.40–1.28)	0.77 (0.43–1.39)
3rd tertile	0.90(0.66–1.22)	1.16 (0.83–1.61)	1.13 (0.88–1.45)	1.21 (0.93–1.57)	1.26 (0.77–2.07)	1.36 (0.81–2.29)
Overcommitment						
1st tertile (low score)	1.0	1.0	1.0	1.0	1.0	1.0
2nd tertile	0.83 (0.62–1.11)	0.91 (0.67–1.23)	0.97 (0.76–1.25)	0.99 (0.76–1.28)	0.99 (0.57–1.72)	1.04 (0.59–1.82)
3rd tertile	0.53 (0.37–0.75)	0.63 (0.43–0.93)	0.85 (0.65–1.12)	0.87 (0.65–1.17)	1.33 (0.77–2.30)	1.46 (0.80–2.63)

95% CI: 95% confidence interval. OR: odds ratio. Adjusted ^A^ crude model + adjusted by total effect variables (age—schooling level—weekly workload—work shift).

**Table 5:** Multinomial logistic regression, with an odds ratio (OR) and confidence interval (CI) of 95%, of the association of psychosocial stress at work, measured by models DC-SS, ERI-OC, and DC-ERI, and adjusted glycemic alterations by selected variables, for active men in the second wave of ELSA-Brazil 2008–2014.

Men	Prediabetes Maintenance		Incident Prediabetes		Incident Diabetes	
	Crude OR (95% CI)	Adj OR ^A^ (95% CI)	Crude OR (95% CI)	Adj OR ^A^ (95% CI)	Crude OR (95% CI)	Adj OR ^A^ (95% CI)
Model 1						
DC and low SS absent	1.0	1.0	1.0	1.0	1.0	1.0
DC present	1.61 (1.06–2.43)	1.44 (0.94–2.19)	1.13 (0.79–1.6)	1.04 (0.73–1.48)	0.99 (0.49–2.02)	0.89 (0.44–1.83)
Low SS present	0.98 (0.65–1.48)	1.11 (0.73–1.69)	0.8 (0.57–1.11)	0.88 (0.63–1.23)	1.26 (0.71–2.26)	1.42 (0.79–2.56)
DC and low SS present	0.98 (0.65–1.48)	1.06 (0.7–1.61)	1.02 (0.75–1.4)	1.07 (0.78–1.47)	0.98 (0.53–1.82)	1.01 (0.54–1.88)
	*p* schooling interaction = 0.3236					
Model 2						
ERI and OC absent	1.0	1.0	1.0	1.0	1.0	1.0
ERI present	0.97 (0.63–1.49)	1.06 (0.69–1.63)	0.95 (0.67–1.35)	0.98 (0.68–1.4)	1.51 (0.84–2.73)	1.59 (0.88–2.87)
OC present	0.99 (0.62–1.57)	0.99 (0.62–1.59)	0.90(0.6–1.33)	0.92 (0.62–1.38)	1.18 (0.58–2.37)	1.21 (0.60–2.47)
ERI and OC present	0.58 (0.37–0.93)	0.65 (0.40–1.05)	0.90(0.65–1.25)	0.98 (0.7–1.37)	0.8 (0.41–1.57)	0.86 (0.43–1.71)
	*p* schooling interaction = 0.2559					
Model 3						
DC and ERI absent	1.0	1.0	1.0	1.0	1.0	1.0
DC present	1.45 (1.02–2.06)	1.35 (0.94–1.94)	1.17 (0.87–1.57)	1.09 (0.81–1.48)	0.91 (0.5–1.63)	0.82 (0.45–1.49)
ERI present	0.88 (0.53–1.44)	0.99 (0.6–1.64)	0.86 (0.58–1.28)	0.91 (0.61–1.37)	1.2 (0.62–2.29)	1.28 (0.67–2.48)
DC and ERI present	0.87 (0.56–1.36)	0.90(0.58–1.41)	1.09 (0.79–1.51)	1.10(0.79–1.53)	0.93 (0.49–1.74)	0.92 (0.49–1.75)
	*p* schooling interaction = 0.5120					

DC: demand–control model. SS: social support. ERI: effort–reward imbalance model. OC: overcommitment. 95% CI: 95% confidence interval. OR: odds ratio. Adjusted ^A^ crude model + adjusted by total effect variables (age—weekly workload—work shift—schooling level).

**Table 6:** Multinomial logistic regression, with an odds ratio (OR) and confidence interval (CI) of 95%, of the association of psychosocial stress at work, measured by models DC-SS, ERI-OC, and DC-ERI, and adjusted glycemic alterations by selected variables, for active women in the second wave of ELSA-Brazil 2008–2014.

Women	Prediabetes Maintenance		Incident Prediabetes		Incident Diabetes	
	Crude OR (95% CI)	Adj OR ^A^ (95% CI)	Crude OR (95% CI)	Adj OR ^A^ (95% CI)	Crude OR (95% CI)	Adj OR ^A^ (95% CI)
**Model 1**						
DC and low SS absent	1.0	1.0	1.0	1.0	1.0	1.0
DC present	1.30(0.88–1.92)	1.08 (0.72–1.62)	1.48 (1.06–2.08)	1.35 (0.96–1.90)	2.59 (1.32–5.08)	2.37 (1.2–4.68)
Low SS present	0.88 (0.6–1.29)	1.02 (0.69–1.53)	1.06 (0.76–1.47)	1.10(0.79–1.54)	1.29 (0.63–2.64)	1.34 (0.65–2.76)
DC and low SS present	1.04 (0.74–1.45)	1.15 (0.81–1.62)	1.46 (1.11–1.94)	1.49 (1.12–1.98)	1.66 (0.89–3.10)	1.70(0.90–3.21)
	*p* schooling interaction = 0.7303					
**Model 2**						
ERI and OC absent	1.0	1.0	1.0	1.0	1.0	1.0
ERI present	1.28 (0.92–1.78)	1.48 (1.06–2.08)	1.24 (0.94–1.64)	1.28 (0.97–1.71)	1.06 (0.57–2.00)	1.12 (0.59–2.13)
OC present	0.62 (0.39–1.01)	0.73 (0.44–1.21)	0.73 (0.50–1.07)	0.76 (0.52–1.12)	0.77 (0.34–1.72)	0.84 (0.37–1.92)
ERI and OC present	0.63 (0.43–0.91)	0.76 (0.51–1.13)	1.01 (0.78–1.32)	1.03 (0.78–1.37)	1.67 (1.03–2.73)	1.82 (1.07–3.07)
	*p* schooling interaction = 0.0371					
**Model 3**						
DC and ERI absent	1.0	1.0	1.0	1.0	1.0	1.0
DC present	1.02 (0.74–1.41)	0.93 (0.67–1.3)	1.34 (1.03–1.75)	1.28 (0.98–1.68)	1.39 (0.78–2.46)	1.33 (0.74–2.37)
ERI present	0.65 (0.40–1.07)	0.81 (0.49–1.36)	0.98 (0.67–1.43)	1.02 (0.70–1.51)	1.03 (0.46–2.33)	1.08 (0.47–2.47)
DC and ERI present	1.14 (0.83–1.58)	1.24 (0.88–1.74)	1.52 (1.16–1.98)	1.51 (1.15–1.99)	2.07 (1.21–3.55)	2.10(1.20–3.65)
	*p* schooling interaction = 0.5658					

DC: demand–control model. SS: social support. ERI: effort–reward imbalance model. OC: overcommitment. 95% CI: 95% confidence interval. OR: odds ratio. Adjusted ^A^ crude model + adjusted by total effect variables (age—weekly workload—work shift—schooling level).

**Table 7:** Multinomial logistic regression, with an odds ratio (OR) and confidence interval (CI) of 95%, of the association of psychosocial stress at work, measured by the combination of model ERI-OC, and adjusted glycemic alterations by selected variables, for active women in the second wave of ELSA-Brasil 2008–2014.

Schooling Interaction Women	Maintenance Prediabetes		Incident Prediabetes		Incident Diabetes	
	Crude OR (95% CI)	Adj OR ^A^ (95% CI)	Crude OR (95% CI)	Adj OR ^A^ (95% CI)	Crude OR (95% CI)	Adj OR ^A^ (95% CI)
**Model 2.1**	**Low Schooling**					
ERI and OC absent	1.0	1.0	1.0	1.0	1.0	1.0
ERI present	1.70(1.02–2.82)	1.87 (1.11–3.13)	1.34 (0.92–1.95)	1.34 (0.91–1.95)	0.64 (0.24–1.68)	0.65 (0.24–1.7)
OC present	1.17 (0.66–2.10)	1.15 (0.63–2.08)	0.81 (0.51–1.27)	0.75 (0.47–1.18)	0.53 (0.18–1.53)	0.51 (0.17–1.48)
ERI and OC present	0.85 (0.51–1.42)	0.83 (0.48–1.41)	0.93 (0.66–1.31)	0.84 (0.59–1.20)	1.12 (0.59–2.10)	1.04 (0.54–2.00)
**Model 2.2**	**High Schooling**					
ERI and OC absent	1.0	1.0	1.0	1.0	1.0	1.0
ERI present	1.09 (0.70–1.68)	1.28 (0.82–1.99)	1.15 (0.75–1.74)	1.21 (0.79–1.85)	1.82 (0.76–4.35)	1.96 (0.81–4.73)
OC present	0.35 (0.12–0.97)	0.27 (0.08–0.87)	0.75 (0.36–1.54)	0.73 (0.35–1.50)	1.68 (0.48–5.94)	1.66 (0.47–5.92)
ERI and OC present	0.82 (0.46–1.46)	0.81 (0.44–1.48)	1.63 (1.04–2.55)	1.96 (0.81–4.73)	4.25 (1.94–9.30)	4.34 (1.96–9.60)

ERI: effort–reward imbalance model. OC: overcommitment. 95% CI: 95% confidence interval. OR: odds ratio. Adjusted ^A^ crude model + adjusted by total effect variables (age—weekly workload—work shift).

## Supplementary Materials

The following are available online at https://www.mdpi.com/1660-4601/17/5/1539/s1, Figure S1: Directed Acyclic Grapg (DAG) representing the association between Occupational Stress and glycemic alterations.
