# Supplementary material for: Combined Use of Job Stress Models and the Incidence of Glycemic Alterations (Prediabetes and Diabetes): Results from ELSA-Brasil Study
Source: Int J Environ Res Public Health. 2020 Feb 27;17(5):1539. doi: 10.3390/ijerph17051539 (PMC7084759; doi:10.3390/ijerph17051539)
Supplement: Supplementary file 1 [file ijerph-17-01539-s001.pdf]

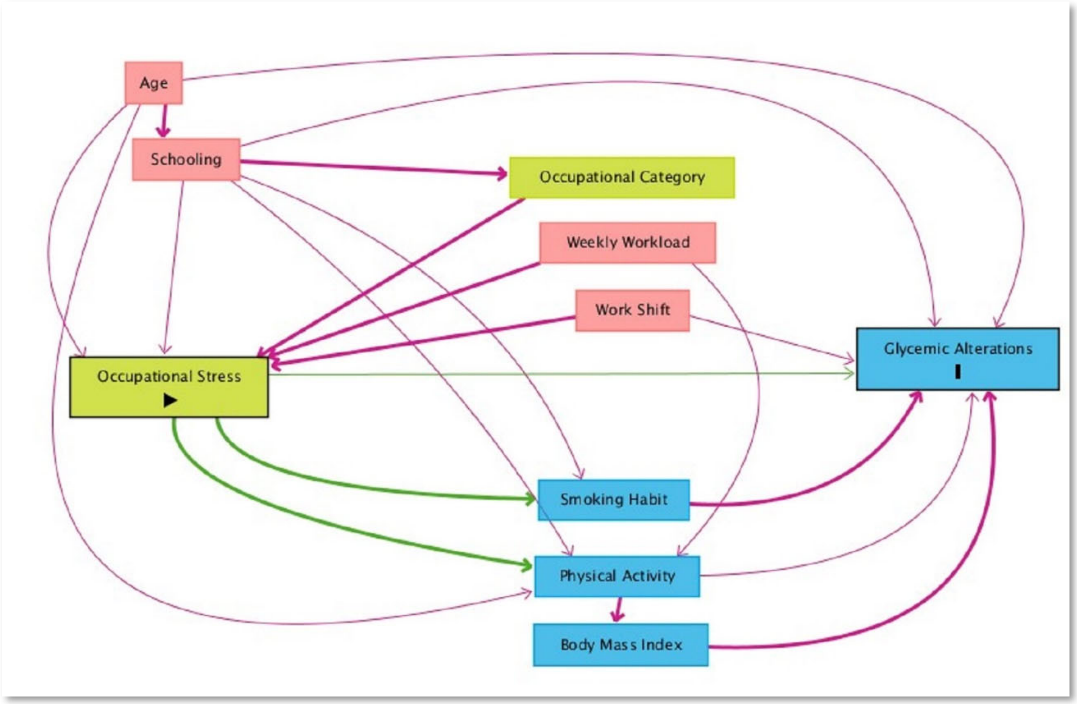

Figure S1. Directed Acyclic Grapg (DAG) representing the association between Occupational Stress and glycemic alterations.

The symbols ► and ▌ denote the exposure and outcome variables, respectively. The pink rectangles represent the variables that simultaneously precede the exposure and the outcome, and the blue rectangles indicate the variables that precede only the outcome. The green lines represent the causal paths (mediators) and the pink lines indicate the paths that potentially skew the estimates of the studied association (confounders).

Minimal sufficient adjustment sets containing Schooling for estimating the total effect of Occupational Stress on Glycemic Alterations: Age, Schooling, Weekly Workload, Work Shift.
